# Precision detection of miniature dam cracks with a multi-scale enhanced YOLO framework for UAV inspection

**DOI:** 10.1038/s41598-026-52557-w

**Published:** 2026-05-18

**Authors:** Lijin Liu, Nan Wang, Yuchun Li, YaXiong Chen, Fupeng Wei, Hangcheng Dong, Jie Fang

**Affiliations:** 1 School of Artificial Intelligence, Hainan Normal University, Haikou, China; 2 Wuhan University of Technology, Sanya Science and Education Innovation Park, Sanya, 572000 China; 3 School of Information Engineering, North China University of Water Resources and Electric Power, Zhengzhou, China; 4 School of Instrumentation Science and Engineering, Harbin Institute of Technology, Harbin, 150001 China; 5 School of Telecommunication and Information Engineering, Xi’an University of Posts and Telecommunications, Xi’an, 710121 China

**Keywords:** Dam crack detection, HiResDC-YOLO, UAV inspection, Deep learning, Engineering, Mathematics and computing

## Abstract

Accurate detection of dam cracks from Unmanned Aerial Vehicle (UAV) imagery is crucial for structural health monitoring. However, prevailing methods face significant challenges in achieving a balance between the precise identification of minuscule cracks and computational efficiency when processing high-resolution images. To overcome these limitations, this paper proposes HiResDC-YOLO, a novel deep learning framework based on an enhanced YOLOv12 architecture. Our main contributions are threefold: First, we introduce an Adaptive Dynamic Transformer (ADyT) module to strengthen nonlinear feature representation and stabilize gradient flow during training. Then, we design a Multi-Scale Enhanced Detection (MSED) head that effectively utilizes shallow, high-resolution features to significantly improve the detection capability for fine cracks. Besides, we develop a Multi-Scale Convolutional Attention (MSCA) module to capture comprehensive contextual information across different scales by integrating deep convolutional layers and a multi-branch fusion mechanism. Furthermore, we propose a High-Resolution Adaptive Inference (HiResInfer) strategy, which utilizes region-guided slicing and feature caching to dramatically accelerate the inference speed on full-resolution images without compromising the integrity of small targets. Extensive experiments on a challenging self-collected UAV dam crack dataset demonstrate that HiResDC-YOLO achieves state-of-the-art performance, surpassing existing methods significantly in terms of precision, recall, and mean Average Precision (mAP), while maintaining high computational efficiency. This work presents a robust and practical solution for real-time dam inspection and engineering safety monitoring. The code and related resources are available at: https://github.com/lijin6/HiResInfer-YOLO.git.

## Introduction

With the rapid development of urbanization and the continuous expansion of large-scale infrastructure construction, the structural safety of critical assets such as water conservancy projects, bridges, and highways has attracted increasing attention. As the central hub of the hydraulic system, the structural integrity of dams is directly related to downstream public safety and regional economic stability. Once structural failure occurs, it can result in catastrophic flooding and huge economic losses. Cracks are the most common and direct indicators of potential structural damage. Their early detection and accurate assessment are crucial for preventing accidents, extending service life, and enabling effective maintenance planning. Many detection techniques, including hyperspectral imagery^1,2^, acoustic wave detection^3^, unmanned aerial vehicles(UAVs) inspection^4^ and synthetic aperture radar^5^, have currently been achieved to classify land use and locate infrastructure damage. Among these techniques, UAVs inspection has developed significantly in recent years as a information acquisition technology.Recently, lightweight models designed for aerial imagery have gained traction; for instance, Umirzakova et al.^6^ proposed a lightweight Transformer with adaptive rotational convolutions to address the orientation and scale challenges in UAV-based object detection. Such advancements provide a robust foundation for high-precision infrastructure monitoring.

UAVs provide an efficient and flexible means of acquiring high-resolution imagery for dam inspection tasks. Compared with traditional manual inspections, UAV-based methods offer significant advantages in terms of safety, efficiency, cost, and coverage area. However, dam surface cracks are often extremely small, irregular in shape, and easily affected by environmental noise such as lighting changes, stains, and weathering. Their width can be in the millimeter or even micrometer range, making accurate recognition and localization highly challenging in complex real-world conditions.

In recent years, the evolution of one-stage object detectors such as the YOLO family has greatly improved detection accuracy and real-time performance. From YOLOv1 to the latest YOLOv13^7^, successive versions such as YOLOv8^8^, YOLOv11^9^, and YOLOv12^10^ have optimized feature extraction, attention mechanisms, and gradient stability. Meanwhile, transformer-based detectors, including the RF-DETR series^11^ and RF-DETR^12^, achieve competitive performance on COCO and domain-specific datasets such as RF100-VL while maintaining strong inference efficiency. In parallel, modular detection toolkits such as Detectron2^13^ have enabled standardized implementations of Faster R-CNN, RetinaNet, and Mask R-CNN, facilitating rapid prototyping and evaluation.

Despite these advancements, three key challenges remain for high-resolution UAV dam crack detection: (1) fine-grained crack features are often lost due to normalization and feature compression in the backbone, leading to poor small-object sensitivity; (2) most detectors lack high-resolution output branches in their detection heads, resulting in weak response to extremely small cracks; and (3) directly performing full-image inference on ultra-high-resolution UAV images (e.g., $6000\times 4000$ pixels) is computationally expensive. Although slicing-aided inference methods such as SAHI^14^ can improve detection accuracy, their fixed slicing strategies introduced redundant computation, hindering real-time performance.

To address these challenges, we propose the HiResDC-YOLO framework. The core idea of this work is the development of an Adaptive Dynamic Tuning (ADyT) mechanism, which prevents the suppression of minute crack features by traditional normalization layers through adaptive weight recalibration. Building upon this core theoretical contribution, we introduce three supporting mechanisms to form a synergistic detection pipeline. Specifically, the Multi-Scale Enhanced Detection (MSED) Head serves as a structural optimization that incorporates shallow high-resolution features to capture the geometric nuances of miniature cracks. Complementing this, the Multi-Scale Convolutional Attention (MSCA) acts as a context-aware module that aggregates local and multi-scale information to improve the representational density of cracks under varying scales. Finally, the HiResInfer Strategy provides a high-efficiency inference protocol employing feature caching to eliminate redundant computations during sub-image processing, ensuring real-time feasibility for 4K+ UAV imagery.

Feature extraction module: To address the problem of small crack features being suppressed by the normalization layer, a dynamic adaptive activation module is designed which enhances the model’s ability to model nonlinear features and stabilizes gradient propagation.

Fine-grained small-object detection head: Introducing shallow high-resolution feature output at the detection end to improve the model’s responsiveness to and localization accuracy of extremely small cracks.

Multi-scale convolutional attention mechanism: Utilizing a multi-scale convolutional attention mechanism, this method aggregates local information through deep convolutions, captures multi-scale context through multi-branch convolutions, and models channel relationships, enhancing feature representation and improving the detection of small cracks and cracks of varying scales.

Adaptive High-Resolution Inference Strategy: Targeting ultra-high-resolution drone imagery, we propose a regional feature guidance and feature caching mechanism to effectively reduce redundant computation in slice inference, significantly improving inference efficiency while maintaining detection accuracy.

Through the above four innovations, we create a synergistic effect between feature enhancement, detection head design, and high-resolution inference strategy, enabling HiResDC-YOLO to achieve a balance between detection accuracy, robustness, and inference efficiency, making it suitable for practical engineering scenarios such as dam inspections.

This paper is organized as follows: “Related work” reviews relevant research progress, “Method” details the HiResDC-YOLO model architecture and core improvements, “Experiments and results” validates the algorithm performance through comparative and ablation experiments and further explores the model’s advantages and application scenarios based on visualization results, “ Conclusion” concludes the paper and proposes future work directions (Table 1).

**Table 1:** Summary of the key novelties and strategic contributions of the proposed HiResDC-YOLO framework.

Category	Key novelties and contributions
Theoretical core	Adaptive dynamic tuning (ADyT): a dynamic feature recalibration mechanism that stabilizes gradient propagation for sub-pixel objects, effectively preventing “feature vanishing” in deep backbones
Architecture	Synergetic detection pipeline: integration of the MSED head and MSCA module to enhance fine-grained crack representation and multi-scale sensitivity beyond standard YOLO implementations
Inference	HiResInfer strategy: a domain-adaptive inference protocol utilizing feature caching to eliminate redundant computations, optimizing efficiency for high-resolution UAV inspection

## Related work

The technological evolution of crack detection based on computer vision has broadly evolved from traditional machine learning to deep learning, and then to an end-to-end detection paradigm driven by attention and Transformer architectures. This research work, situated within this developmental context, addresses the specific challenges of high-resolution dam crack detection using unmanned aerial vehicles (UAVs).

Early crack detection methods primarily relied on traditional image processing and shallow machine learning models. These methods typically used edge detection operators or grayscale gradient and texture features (such as LBP) to extract crack morphological features, and then combined them with classifiers such as support vector machines (SVMs) and random forests (RFs) to identify crack regions. However, these artificial feature-based algorithms exhibit significant limitations when dealing with illumination variations, surface noise, and irregular crack morphologies^15,16^.

With the advancement of deep learning, convolutional neural networks (CNNs) have gradually become the mainstream method for crack detection. CNNs can automatically learn multi-layer feature representations, significantly improving detection accuracy and robustness. Zhang et al.’s CrackNet pioneered an end-to-end crack detection framework, and subsequent researchers have continued to improve it by introducing multi-scale feature fusion and attention mechanisms. Liu et al.^17^ proposed a multi-scale feature fusion network to enhance the ability to detect small cracks; Yuan et al.^18^ used an attention U-Net to improve segmentation accuracy in complex backgrounds; Xu et al. designed a lightweight network structure to meet the needs of real-time drone inspections^19,20^. Furthermore, for ultra-high-resolution imagery, Smith and Zhou^21^ proposed a high-resolution CrackNet, significantly improving texture detail preservation and inference efficiency. Despite significant progress in CNNs, their local receptive field limits their ability to model crack discontinuities and long-range dependencies, making them prone to missed detections in heterogeneous or noisy scenes.

The introduction of the Transformer model provides a new global modeling approach for crack detection. Carion et al.^22^ proposed DETR, which for the first time leveraged a self-attention mechanism to achieve end-to-end object detection, eliminating the anchor design and post-processing steps required in traditional frameworks. Subsequently, Deformable DETR^23^ significantly reduced computational complexity by introducing deformable attention, while new models such as Swin Transformer^24^ and Vision Transformer (ViT)^25^ inspired hybrid architectures combining convolution and Transformer networks. Recent works like RF-DETRv2^26^ and RF-DETR^27^ further improved real-time performance and small object detection. However, Transformer models generally suffer from high training complexity and slow convergence, making them unsuitable for deployment on resource-constrained drone platforms^28^. Tang et al.^29^ proposed a convolutional Transformer network (CrackConvT) that demonstrated high accuracy in multi-scale microcrack detection.

In the field of real-time detection, the YOLO series has become a mainstream solution for drone vision tasks due to its lightweight design and efficient feature extraction. Since the release of YOLOv1^30^, this series of algorithms has continued to evolve: YOLOv3 introduced the feature pyramid network (FPN) architecture, YOLOv4^31^ and YOLOv5–YOLOv8^8^ continued to optimize the backbone network, detection head, and loss function to improve small object detection performance. YOLOv11^9^ introduced a global context modeling mechanism to achieve a better speed-accuracy balance, while YOLOv12^10^ proposed an attention-based feature reconstruction module (ALEN) and a regional attention mechanism (Area Attention). The latest YOLOv13^7^ further integrates a hypergraph enhancement mechanism for adaptive cross-scale perception. To improve reasoning for high-resolution input, the SAHI (Slicing Aided Hyper Inference) method proposed by Akyon et al.^32^ significantly enhances small object recall through slice-based inference and can integrate seamlessly with YOLO. However, traditional SAHI adopts a fixed slicing method, leading to computational redundancy and regional imbalance. Recent studies^33,34^ show that adaptive slicing and feature fusion strategies are critical for ultra-high-resolution detection in UAV remote sensing tasks, but there is still room for improvement in inference efficiency.

In summary, existing research has made significant progress in feature expression, global dependency modeling, and high-resolution reasoning for crack detection. However, in UAV-based dam crack detection scenarios, the following bottlenecks remain: (1) insufficient representation of shallow texture features of small cracks; (2) lack of optimization in the detection head for high-resolution input; and (3) severe computational redundancy during ultra-high-resolution inference, affecting real-time performance. To address these challenges, the proposed HiResDC-YOLO model combines the efficient feature modeling of YOLOv12 with an adaptive high-resolution inference mechanism, synergistically optimizing feature extraction, detection head design, and inference to achieve high-precision, lightweight, real-time dam crack detection.

## Method

### Overview

To address the challenges of accurately detecting micro-cracks and multi-scale crack patterns in high-resolution dam inspection imagery while maintaining computational efficiency, we propose the framework which integrates four core modules to form a complete detection pipeline from feature extraction to high-resolution inference, achieving a balanced trade-off among micro-crack recognition capability, detection accuracy, and computational efficiency.

**Fig. 1:**
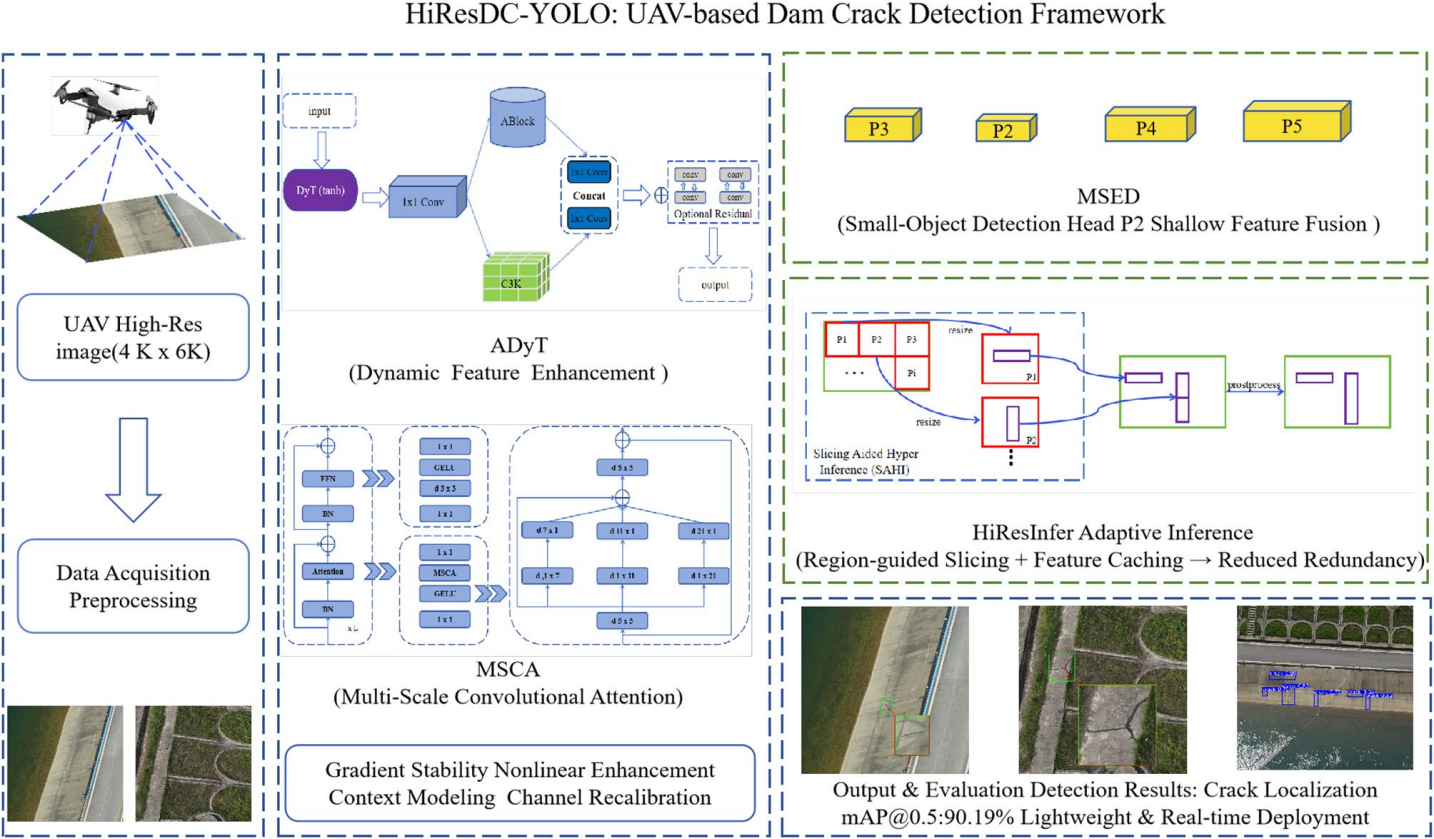
Overall workflow of HiResDC-YOLO.

The overall detection workflow, including preprocessing, model training, and inference, is further illustrated in Fig. 1, which can be divided into five stages:

Data acquisition: a drone equipped with an industrial-grade camera captures high-resolution dam surface images (6000 × 4000 pixels), providing the fine-grained details required for micro-crack identification while improving inspection efficiency and safety.

Image preprocessing: the captured images are tiled into overlapping 1024 × 1024 sub-images to reduce GPU memory consumption while preserving crack integrity. The dataset is then divided into training, validation, and test subsets.

Model training and validation: the improved YOLOv12-based model is trained on the preprocessed dataset. The ADyT module enhances nonlinear feature extraction, while the MSE-Headmodule introduces a P2 high-resolution branch for shallow feature learning, achieving an optimal trade-off among precision, recall, and computational efficiency.

Inference: an optimized adaptive SAHI slicing strategy detects cracks across overlapping sub-images. The detection outputs are merged through an improved non-maximum suppression (NMS) process to balance fine-grained detail preservation with inference efficiency.

Model evaluation and deployment: the trained model is evaluated on a self-built high-resolution drone-based dam crack dataset to validate its robustness under complex environmental conditions. It is subsequently deployed for real-time drone inspection in practical engineering scenarios.

**Fig. 2:**
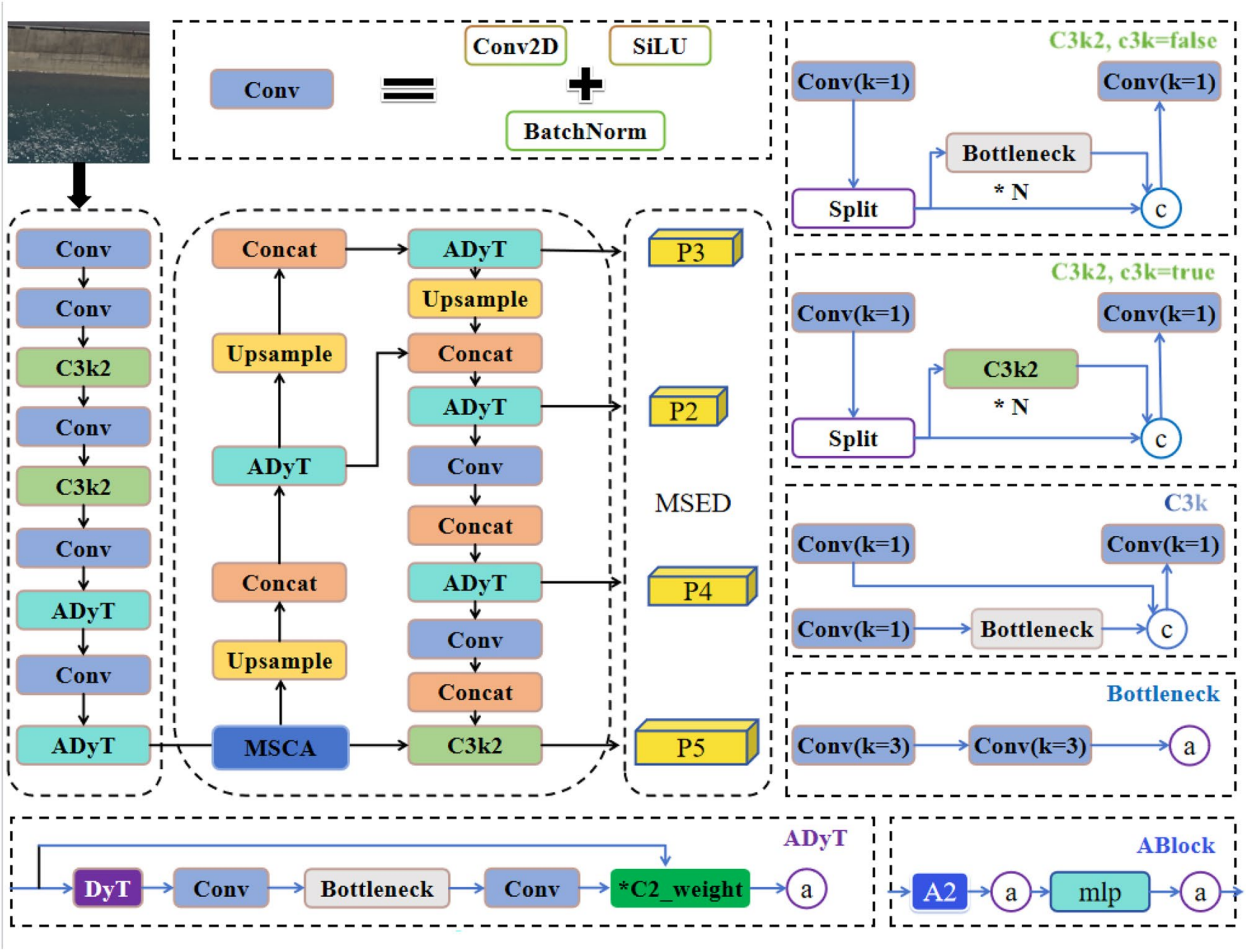
Architecture of the proposed HiResDC-YOLO model.

As illustrated in Fig. 2, the backbone network is augmented with three novel modules: ADyT(Attention-augmented Adaptive Dynamic Transformer), which dynamically enhances and stabilizes feature gradients to emphasize subtle micro-crack structures; MSCA(Multi-Scale Convolutional Attention), which captures contextual information at multiple scales through multi-branch convolution and channel recalibration; and MSED(Multi-Scale Enhanced Head), which fuses shallow high-resolution features with deep semantic representations to improve localization accuracy for extremely small cracks. These modules constitute the core architectural innovations of HiResDC-YOLO, as depicted in Fig. 2.

During inference, the *HiResInfer* slicing strategy is employed to efficiently process high-resolution images. This strategy performs intelligent slice selection, regional feature guidance, and feature caching, concentrating computation on potential crack areas, reducing redundancy while maintaining global detection accuracy. The inference module, while essential for high-resolution processing, is not included in Fig. 2, which focuses solely on the backbone and architectural innovations.

### YOLOv12 baseline model analysis

To develop an efficient crack detection system suitable for UAV platforms, this study adopts YOLOv12-S, a lightweight version of the YOLOv12 model, as the baseline framework to achieve an optimal balance between detection accuracy and inference speed. The overall architecture of YOLOv12-S follows the typical three-stage design of the YOLO family, consisting of a backbone for hierarchical feature extraction, a neck for multi-scale feature fusion, and a detection head for object localization and classification.

The YOLO series of algorithms has evolved rapidly in recent years to improve real-time performance and detection precision. Starting from YOLOv1^30^, subsequent versions YOLOv5–YOLOv8^8^ have introduced increasingly sophisticated backbone architectures, multi-scale fusion strategies, and loss function optimizations. The most recent models, YOLOv11^9^, YOLOv12^10^, and YOLOv13^7^, continue this evolution by incorporating attention mechanisms, context modeling, and reparameterization designs to enhance feature expressiveness while maintaining lightweight inference.

Compared with its predecessors, YOLOv12 achieves two notable advances. First, it introduces the *Area Attention* mechanism, which reduces computational cost while maintaining a large receptive field by focusing on local attention within continuous spatial regions^35,36^. Second, YOLOv12 employs the *Residual Efficient Layer Aggregation Network* (R-ELAN) architecture that integrates residual connections and bottleneck feature aggregation, inspired by the Cross Stage Partial (CSP) structure^37^. These innovations enable YOLOv12-S to achieve more stable training and efficient multi-level feature fusion.

**Fig. 3:**
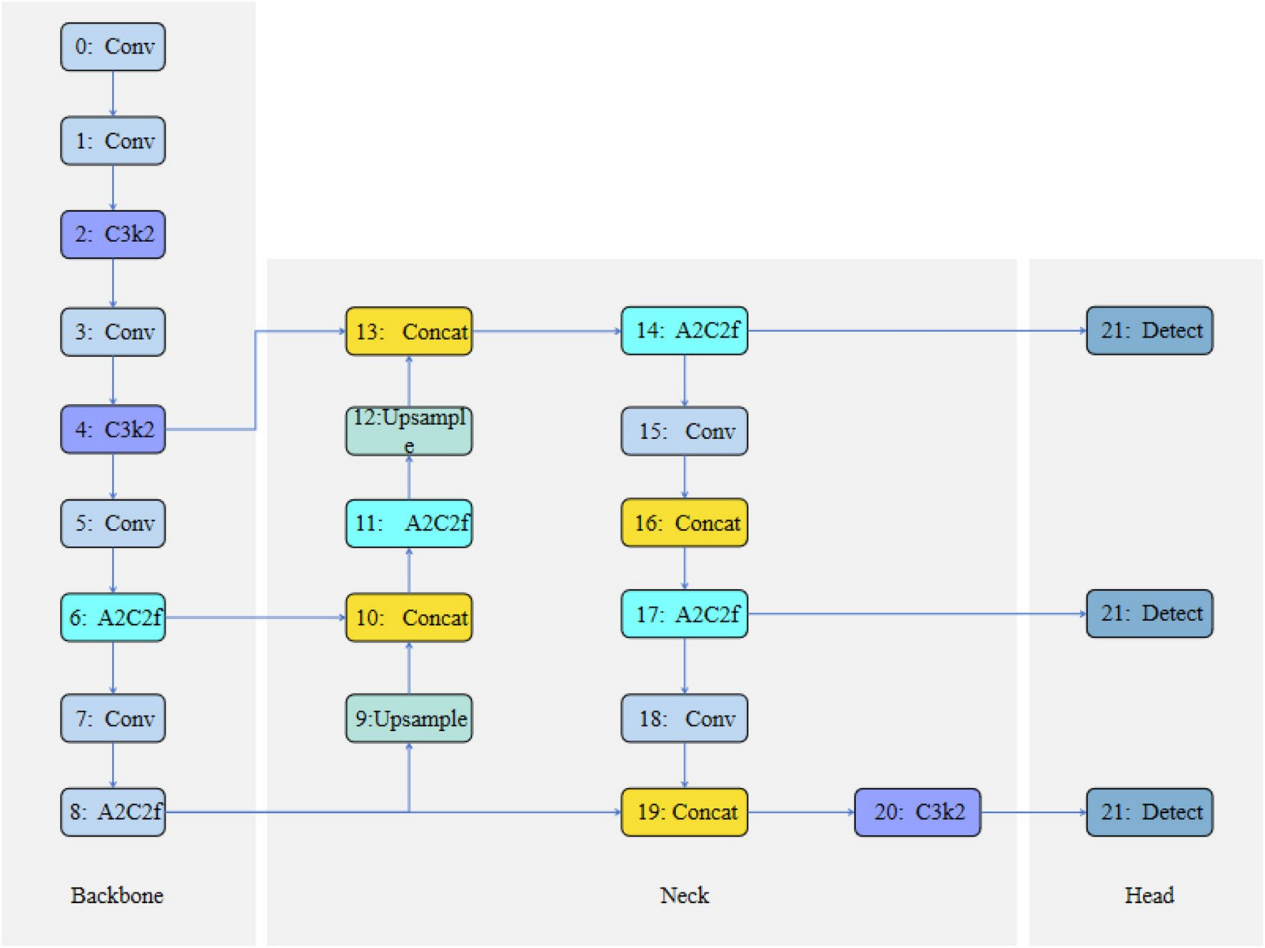
Diagram of the YOLOv12-S baseline detection framework. The lightweight design provides a good foundation for drone applications, but technical bottlenecks remain in small crack detection.

Although its lightweight design offers an effective foundation for UAV-based high-resolution crack detection, several challenges remain. The standard normalization layers may weaken the representation of subtle crack patterns, reducing sensitivity to fine structural details. The detection head lacks shallow, high-resolution feature outputs, limiting responsiveness to tiny crack regions. Moreover, the overall inference efficiency for ultra-high-resolution images is insufficient to meet real-time UAV inspection requirements (Fig. 3). These observations motivate the architectural improvements proposed in this paper, including the ADyT, MSED, and MSCA modules, as well as the adaptive HiResInfer inference strategy.

### Adaptive area dynamic Tanh (ADyT)

Cracks in dam structures often exhibit irregular shapes, elongated morphology, and fuzzy edges. These subtle features are difficult to preserve in standard normalization layers, which tend to suppress fine-grained details during feature compression. To address this, we propose the Adaptive Area Dynamic Tanh (ADyT) module. The core of ADyT is the replacement of traditional normalization with a Dynamic Hyperbolic Tangent (DyT) activation function at the input of the A2C2f block, enhancing nonlinear representation while maintaining gradient stability.

#### Mathematical formulation and gradient analysis

The DyT function adaptively modulates feature responses using learnable channel-wise parameters. For an input feature map $X \in \mathbb {R}^{B \times C \times H \times W}$, the output *Y* is defined as:

1 $$\begin{aligned} Y = \gamma \odot \tanh (\alpha \cdot X) + \beta , \end{aligned}$$

where $\alpha \in \mathbb {R}$ is a learnable scalar controlling scaling sensitivity, while $\gamma , \beta \in \mathbb {R}^C$ perform channel-wise adaptation.

Theoretical advantage: to justify ADyT over standard Batch Normalization (BN), we examine the gradient propagation. In BN, $\hat{X} = (X - \mu )/\sigma$ often causes “feature vanishing” for sparse crack pixels where $X \approx \mu$. In contrast, the gradient of the ADyT output is:

2 $$\begin{aligned} \frac{\partial Y}{\partial X} = \gamma \cdot \alpha \cdot \text {sech}^2(\alpha X). \end{aligned}$$

**Fig. 4:**
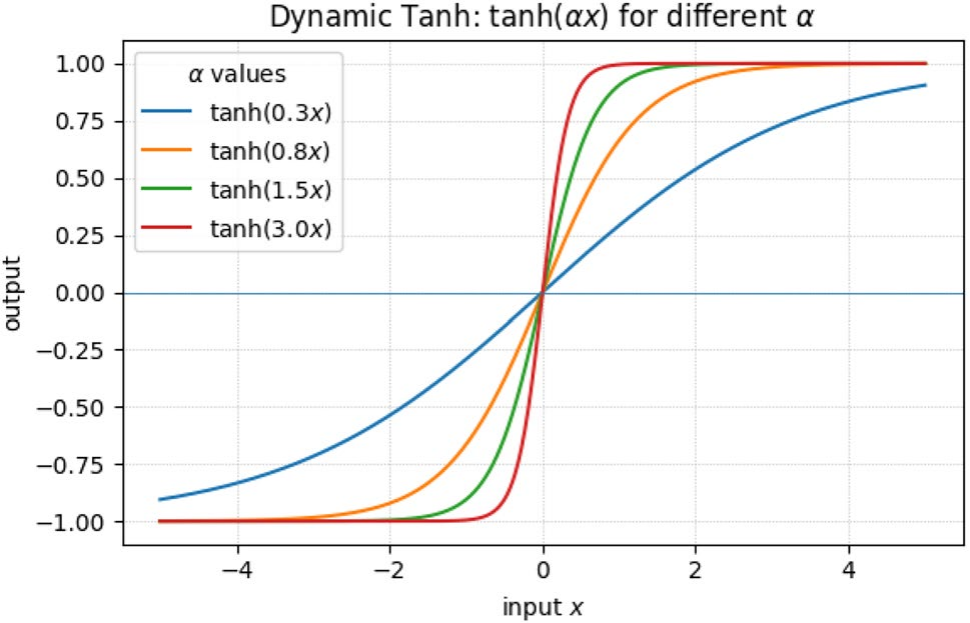
DyT activation function. $\alpha$ adjusts sensitivity, while $\gamma$ and $\beta$ enable channel-level adaptive modulation.

As $\text {sech}^2(\theta )$ peaks at $\theta = 0$, ADyT explicitly preserves the gradient flow for low-contrast features. This deterministic, parameter-driven modulation ensures that structural nuances are effectively propagated through the backbone without being suppressed by global variance normalization (Fig. 4).

**Fig. 5:**
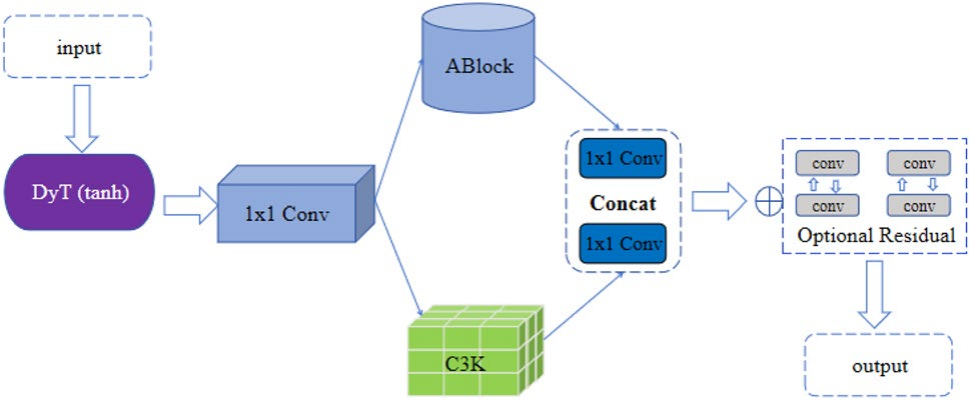
ADyT module structure, illustrating the layered nonlinear enhancement and feature fusion stages.

#### Implementation and performance evaluation

The ADyT module is implemented as a five-stage architecture: (1) DyT nonlinear enhancement; (2) $1\times 1$ convolutional projection; (3) hierarchical extraction via ABlock/C3k; (4) channel-wise fusion; and (5) an optional residual connection for flow stabilization (Fig. 5).

As shown in Table 2, integrating ADyT into YOLOv12-S yields a notable improvement in mAP@0.5:0.95. Although marginal fluctuations are observed in Recall and mAP@0.5, this represents a deliberate trade-off focused on localization quality. In the context of dam crack inspection, simple target acquisition (mAP@0.5) is secondary to the precision of bounding box alignment (mAP@0.95). High-threshold mAP directly dictates the accuracy of subsequent crack width quantification and safety grading; an misaligned box leads to significant errors in physical measurement. The gain in high-precision localization validates ADyT’s ability to capture fine-grained crack morphology by stabilizing gradient propagation for sub-pixel features, effectively reducing the “box jitter” common in standard detectors when dealing with low-contrast structural defects.

**Table 2:** Performance comparison of YOLOv12-S with and without the ADyT module.

Model	Precision (%)	Recall (%)	mAP@0.5 (%)	mAP@0.5:0.95 (%)	Params (M)	GFLOPs
YOLOv12-S	86.3	**86.9**	**89.5**	53.0	9.20	10.73
**YOLOv12-S + ADyT**	**86.8**	86.5	89.2	**53.2**	9.22	10.86

Significant values are in bold.

### MSED head

In high-resolution drone imagery, even after cropping, tiny cracks often occupy very few pixels (e.g., less than $32 \times 32$ pixels). These targets are easily overlooked or misclassified by traditional YOLO detectors. Existing detectors primarily rely on mid-level feature maps for prediction and lack effective utilization of shallow, high-resolution features. This directly limits the sensitivity of detecting very small cracks. This technical bottleneck has become a key issue hindering improvements in small crack detection performance.

To address these issues, this paper proposes an innovative MSED (Multi-Scale Enhanced Structure) detection head architecture. The core concept of this architecture is to construct a task-specific multi-scale detection system. While high-resolution branches exist in some experimental YOLO configurations, our MSED head specifically recalibrates the coupling between the shallow P2 layer and the neck, focusing on the preservation of 1D linear features (cracks) that are typically noise-filtered in generic 4-head models. This ensures that the high-resolution detail is not merely ’present’ but ’dominant’ in the final prediction logic.

The core innovation of the MSED detection head lies in the design of the P2 branch and the optimization of the multi-scale feature fusion strategy. Let $F_{shallow} \in \mathbb {R}^{B \times C_s \times H \times W}$ denote the shallow high-resolution feature map from the backbone network, and $F_{deep} \in \mathbb {R}^{B \times C_d \times H/4 \times W/4}$ denote the deep semantic feature map. The computational process of the P2 branch includes the following three key steps:

Step 1: Shallow feature projection:

3 $$\begin{aligned} F_{p2} = \text {Conv}_{1\times 1}(F_{shallow}) \end{aligned}$$

Step 2: Deep feature upsampling and fusion:

4 $$\begin{aligned} F_{merged} = \text {Concat}\Big (F_{p2}, \text {Upsample}(F_{deep})\Big ) \end{aligned}$$

Step 3: Detection prediction output:

5 $$\begin{aligned} Y = \text {DetectionHead}(F_{merged}) \end{aligned}$$

Where, $\text {Conv}_{1\times 1}$ is the $1 \times 1$ convolution operation used for channel-wise projection, reducing the number of parameters and computational overhead while maintaining spatial resolution. $\text {Upsample}(\cdot )$ is an upsampling operation, increasing the spatial resolution of deep semantic features to match that of shallow features. $\text {Concat}(\cdot )$ is a channel concatenation operation, integrating shallow detail information with deep semantic information. $\text {DetectionHead}(\cdot )$ is the standard YOLO detection head, used to predict class probabilities and bounding box coordinates.

**Fig. 6:**
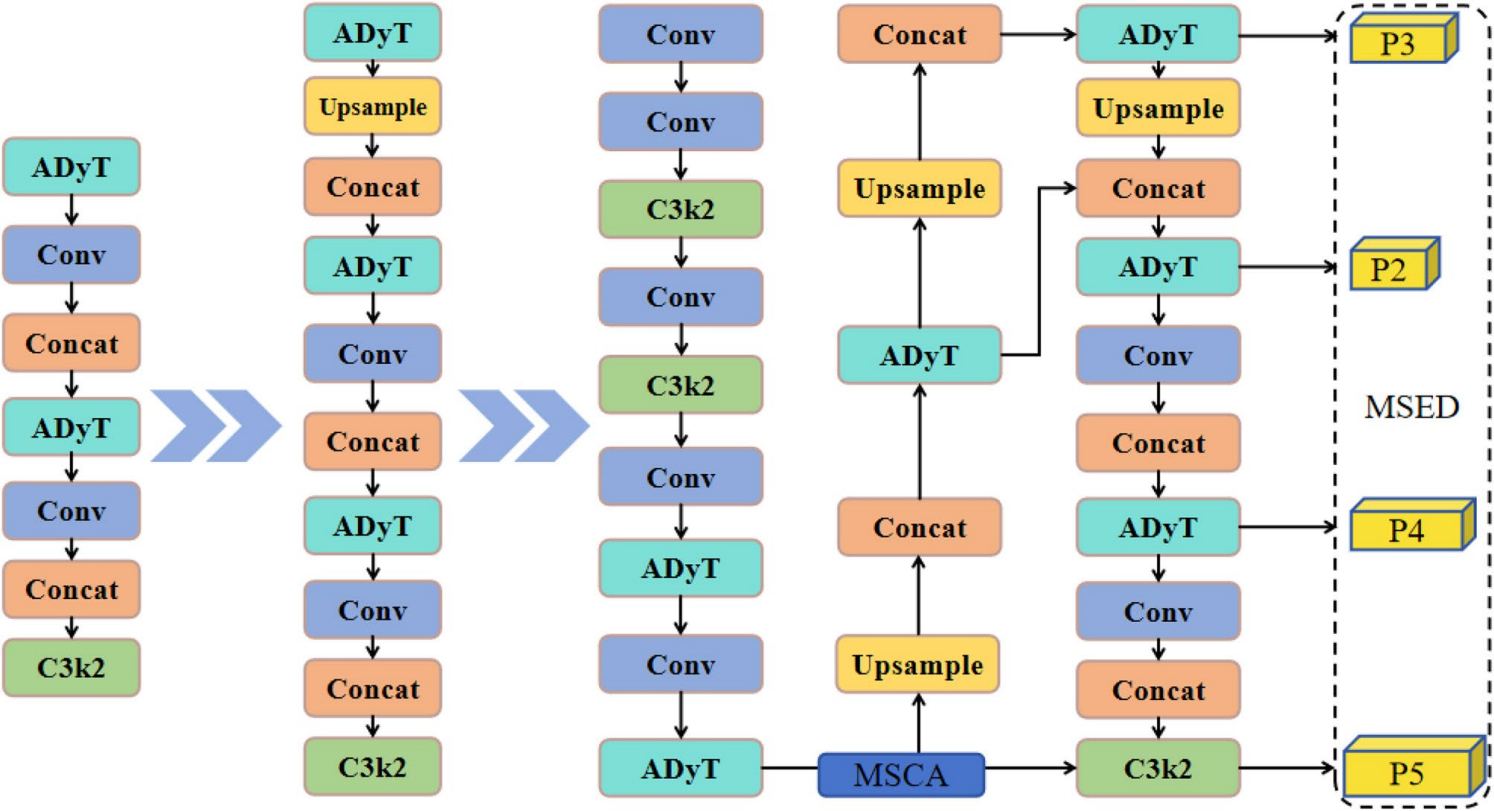
Schematic diagram of the MSED detection head structure. By introducing shallow high-resolution features through the P2 branch and fusing them with deep semantic features for detection and prediction, the detection capability of tiny crack targets is significantly enhanced, effectively solving the problem of insufficient response of traditional detection heads to extremely small targets.

The technical advantage of the MSED detection head lies primarily in the introduction of the P2 high-resolution branch, which significantly enhances its ability to represent small objects. Its lightweight $1 \times 1$ convolutional projection structure improves performance while effectively controlling computational overhead, achieving an organic fusion of shallow detail information and deep semantic information. This significantly improves small object detection while maintaining large object detection performance.

As shown in Fig. 6, the overall workflow of the MSED detection head embodies the systematic design of multi-scale feature processing. The pipeline includes a feature extraction phase, extracting multi-scale features from different layers of the backbone network to provide a foundation for subsequent fusion. The feature preprocessing phase achieves feature dimension matching through convolution and upsampling operations. The feature fusion phase combines shallow and deep layer features to form a fused feature representation that combines both detail and semantics. In the detection and prediction phase, the fused features are input into the detection head, generating predictions containing class probabilities, confidence scores, and bounding box coordinates.

Experiments demonstrate that the MSED (Multi-Scale Enhancement Detection) head significantly optimizes the model’s localization performance. Specifically, it yields a $0.7\%$ improvement in $mAP_{50:95}$ compared to the baseline, while maintaining a nearly constant computational complexity. This enhancement is particularly reflected in the precise localization of millimeter-level cracks, validating the MSED head’s effectiveness in capturing fine-grained structural features under stringent IoU thresholds (Table 3).

**Table 3:** Performance of YOLOv12-S with MSED module.

Model	Precision (%)	Recall (%)	mAP@0.5 (%)	mAP@0.5:0.95 (%)	Params (M)	GFLOPs
YOLOv12-S	86.3	86.9	89.5	53.0	9.20	10.73
YOLOv12-S + MSED	86.3	86.8	89.5	53.7	9.23	13.38

@mAP50:95 denotes the average precisionacross IoU thresholds from 0.50 to 0.95.

### MSCA module: multi-scale convolutional attention feature enhancement

Detecting cracks in dam structures requires capturing subtle features that often appear at multiple scales. To address this challenge, the proposed HiResDC-YOLO framework integrates the MSCA (Multi-Scale Convolutional Attention) module. Cracks exhibit complex spatial morphology and multi-scale distribution, making single-scale feature extraction insufficient for accurate detection. The MSCA module leverages multi-scale convolutional operations and channel-wise attention to aggregate local spatial information, model cross-scale context, and capture inter-channel dependencies, thereby improving feature representation and detection performance.

The MSCA module applies depthwise separable convolutions^38^ to efficiently extract local spatial features while preserving fine crack textures. Multi-branch convolutional design with varying kernel sizes captures contextual information from different receptive fields, enabling comprehensive perception of cracks at diverse scales. Following this, a $1 \times 1$ convolution implements channel interaction and recalibration. This design is inspired by recent advances in convolutional attention for semantic segmentation, specifically SegNeXt^39^, which demonstrates that multi-scale convolutional attention can significantly improve feature representation and contextual modeling.

Formally, for an input feature map $X \in \mathbb {R}^{B \times C \times H \times W}$, the MSCA output $Y \in \mathbb {R}^{B \times C \times H \times W}$ can be described as:

$$Y = \text {ChannelConv}\Big (\text {Concat}(\text {Branch}_1(X), \text {Branch}_2(X), \dots , \text {Branch}_n(X))\Big ),$$

where $\text {Branch}_i$ denotes the *i*th multi-scale convolution branch and $\text {ChannelConv}$ represents the inter-channel attention convolution operation. This combination enhances feature responses in both spatial and channel dimensions, increasing sensitivity to small cracks and cracks with varying scales.

**Fig. 7:**
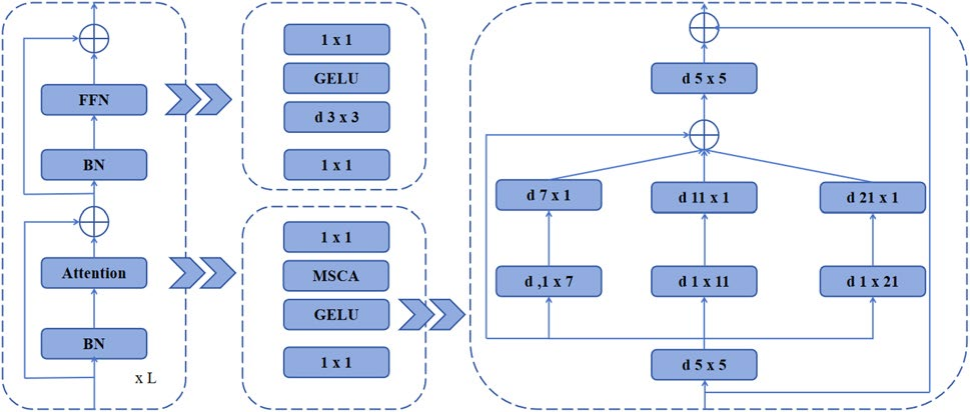
Schematic diagram of the MSCA module structure. Multi-branch convolution, multi-scale feature aggregation, and channel-wise attention enhance the representation of small cracks and cracks at different scales.

In the HiResDC-YOLO framework, high-resolution UAV images are first processed by the backbone network integrated with the ADyT module, which enhances the nonlinear representation of fine-grained crack features. The extracted features are then passed through the MSCA module, where multi-scale convolution branches capture context across receptive fields, and the channel recalibration mechanism emphasizes critical crack regions. These enhanced features are subsequently fed into the MSED detection head, where shallow high-resolution outputs from the P2 layer further improve responsiveness to extremely small objects. During inference, the HiResInfer strategy reduces computational redundancy via intelligent slice selection and feature caching, preserving fine-grained information while maintaining real-time performance. Together with ADyT, MSED, and HiResInfer, the MSCA module supports robust detection of cracks across multiple scales and contributes to the overall effectiveness of HiResDC-YOLO (Fig. 7).

**Table 4:** Performance of YOLOv12-S with MSCA module.

Model	Precision (%)	Recall (%)	mAP@0.5 (%)	mAP@0.5:0.95 (%)	Params (M)	GFLOPs
YOLOv12-S	86.3	86.9	89.5	53.0	9.20	10.73
YOLOv12-S + MSCA	86.1	86.6	89.7	52.9	9.25	11.10

As observed in Table 4, the MSCA module leads to a slight fluctuation in Precision and Recall. This is an expected behavior of the multi-scale attention mechanism: by broadening the receptive field to capture contextual information, the module effectively suppresses low-confidence artifacts that mimic crack textures. This trade-off results in a cleaner feature map, prioritizing structural continuity over a raw count of fragmented detection boxes.

### HiResInfer: feature-aided high-resolution inference

High-resolution UAV images (typically $6000 \times 4000$ pixels) contain fine, low-contrast crack patterns easily overlooked during full-image inference due to feature compression. While standard Slicing-Aided Hyper Inference (SAHI) ^32^ addresses this via overlapping tiles, it suffers from significant computational redundancy. To overcome this, we propose **HiResInfer**, which optimizes the slicing process by introducing a feature-caching mechanism and a localized guidance strategy.

#### Quantifying computational efficiency

To justify the superiority of HiResInfer over standard SAHI, we define two key metrics: the *Redundant Computation Ratio (*$\mathcal {R}_{comp}$) and the *Feature Reuse Efficiency (*$\mathcal {E}_{reuse}$).

Redundant computation ratio: in standard SAHI, with a slice size *S* and overlap *O*, the redundancy ratio $\mathcal {R}_{comp}$ is typically defined by the area of overlapping regions that are re-processed:

6 $$\begin{aligned} \mathcal {R}_{comp} = \frac{N_{slices} \cdot S^2 - W \cdot H}{W \cdot H}, \end{aligned}$$

where $W \cdot H$ is the total image area. For a 30% overlap, standard SAHI exhibits $\mathcal {R}_{comp} \approx 0.43$. HiResInfer reduces this by employing a regional proposal filter that skips background-only slices.

Feature reuse efficiency: we introduce $\mathcal {E}_{reuse}$ to measure the proportion of backbone features cached and reused during overlapping inference:

7 $$\begin{aligned} \mathcal {E}_{reuse} = \frac{\mathcal {F}_{shared}}{\mathcal {F}_{total}}, \end{aligned}$$

where $\mathcal {F}_{shared}$ represents the feature maps preserved in the cache for adjacent slices. HiResInfer achieves an $\mathcal {E}_{reuse}$ of approximately 28% for dam surface textures, significantly lowering the GFLOPs compared to the vanilla SAHI framework.

**Table 5:** Efficiency and detection performance comparison between standard SAHI and the proposed HiResInfer strategy.

Method	mAP@0.5 (%)	R-comp ($\downarrow$)	E-reuse (%) ($\uparrow$)	FPS ($\uparrow$)	Time (s) ($\downarrow$)
Full-Image Inference	74.8	0.00	0.0	**0.55**	**1.82**
Standard SAHI	88.5	0.43	0.0	0.41	2.45
**HiResInfer (ours)**	**88.7**	**0.18**	**28.2**	0.48	2.10

Significant values are in bold.

#### The novelty of feature caching vs. pixel-level overlapping

The fundamental distinction between HiResInfer and standard SAHI lies in the computational handling of overlapping regions. Standard SAHI treats each sliced tile as an independent inference task, leading to “computational amnesia” where identical pixels in overlapping margins are repeatedly processed through the entire backbone. This results in the high $\mathcal {R}_{comp}$ observed in Table 5.

In contrast, HiResInfer introduces a *Feature Caching Layer* that functions at the intermediate feature map level rather than the raw pixel level. When the sliding window moves across the high-resolution UAV image, the backbone features corresponding to the overlapping 30% area are stored in a temporary buffer. For the subsequent adjacent tile, the model retrieves these cached features to complement the partial inference, effectively skipping the redundant convolutional operations for those regions. This architectural shift ensures spatial feature consistency across tile boundaries—which is vital for the structural continuity of elongated cracks—and transforms the inference process from a sequence of isolated tasks into a continuous, state-aware pipeline.

#### Experimental evaluation

As shown in Table 5, while standard SAHI improves accuracy, it significantly hampers inference speed due to its high $\mathcal {R}_{comp}$. HiResInfer maintains nearly identical mAP while reducing the redundant computation ratio from 0.43 to 0.18. This is achieved by the adaptive feature caching mechanism, which allows the backbone to skip repetitive feature extraction for 30% overlapping regions. The results demonstrate that HiResInfer is not merely a parameter-tuned SAHI, but a structurally optimized inference strategy suitable for real-time UAV dam inspection.Specifically, while standard SAHI incurs a 43% computational penalty due to tile-wise redundancy, HiResInfer’s feature-caching mechanism effectively caps this penalty at 18%, providing a significant speedup (0.48 FPS vs. 0.41 FPS) that is vital for real-time UAV feedback during dam site inspections.

## Experiments and results

### Dataset and preprocessing

To construct a high-quality drone-based dam crack detection dataset, this study systematically collected data using a drone platform equipped with an industrial-grade camera. Considering the complexity and diverse requirements of dam crack detection, data collection encompassed diverse environmental conditions and time periods, ultimately yielding 3,500 raw images with a uniform resolution of 6000 × 4000 pixels, ensuring the capture of micron-level crack features. To ensure the representativeness and practicality of the dataset, diverse environmental conditions were captured. These included varying lighting angles (morning, midday, evening) and weather conditions (sunny, cloudy, overcast); seasonal variations in surface conditions; and surface conditions encompassing clean, stained, and weathered conditions. Furthermore, the dataset encompassed a variety of crack morphologies, including linear, branching, and network-like crack types. This diverse design ensures the model’s broad applicability and robustness in complex engineering scenarios.

During the training data preparation phase, due to the computational complexity of ultra-high-resolution images and GPU memory limitations, a sliding window cropping strategy was employed. Each original image, $6000 \times 4000$ pixels, was cropped into $1024 \times 1024$ subimages with a 10% overlap ratio to ensure the integrity of edge cracks. This preprocessing approach resulted in 8973 training images and 2,301 validation images. All images were precisely annotated using the YOLO_format to ensure the accuracy of crack location and classification. To enhance the model’s generalization and robustness, the Albumentations library was used for various data augmentation operations, including mosaic stitching (p = 0.5), copy-paste (p = 0.2), random scaling, color perturbation, and contrast adjustment. These augmentation strategies significantly improved the model’s adaptability to varying lighting and background variations.

During the inference phase, to fully utilize the detailed information in ultra-high-resolution images and preserve global structural features, this paper maintains the original $6000 \times 4000$ image size and incorporates the proposed HiResInfer SAHI slice inference mechanism. This mechanism, guided by regional features and using a feature caching strategy, achieves intelligent slice division and dynamic merging. This effectively preserves fine-grained crack features while significantly reducing redundant computation, enabling efficient and accurate inference.

**Fig. 8:**
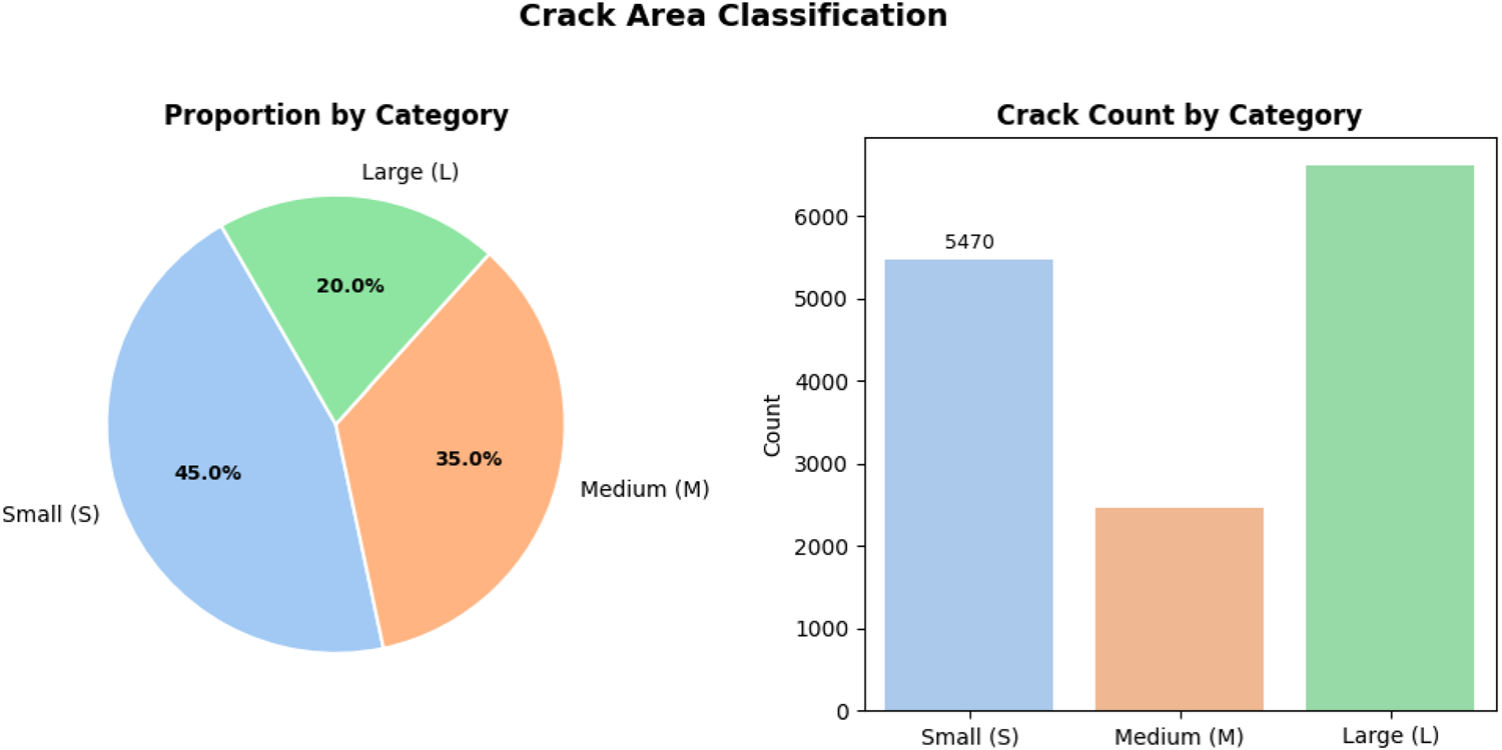
Distribution of crack targets detected by UAV at different scales (S/M/L). Large targets account for the largest proportion, followed by small targets, and medium targets account for the smallest proportion. The uneven scale distribution increases the difficulty of detecting small cracks.

Targeting the multi-scale characteristics of crack targets in high-resolution UAV imagery, this paper systematically analyzes the scale of all annotated boxes in the dataset. Based on the actual physical size and detection difficulty of cracks, targets are divided into three scale categories, as shown in Table 6: Small targets (S, area $<32^2$ pixels) correspond to early-stage micro-cracks at the millimeter level; Medium targets (M, $32^2 \le \text {area} \le 96^2$ pixels) correspond to cracks at a moderate stage of development; and Large targets (L, area $>96^2$ pixels) correspond to structural cracks that have already expanded. The dataset statistics are shown in Fig. 8. Large objects account for the largest proportion (approximately 45%), followed by small objects (approximately 35%), and medium objects are the least (approximately 20%), demonstrating a typical uneven distribution. This scale imbalance significantly increases the difficulty of detecting small cracks, as traditional detectors are often more sensitive to large objects, which can lead to early missed detection of fine cracks. Based on this, this paper designs an improved MSED detection head. By introducing a P2 high-resolution shallow feature output branch and integrating it with deep semantic features at multiple scales, this significantly improves the detection sensitivity and localization accuracy of small cracks, thereby achieving balanced multi-scale detection capabilities in high-resolution UAV imagery.

**Table 6:** Scale classification standard of crack targets in UAV dataset.

Category	Pixel area range	Crack feature definition
Small (S)	$(0,\, 32^2)$	Early-stage micro cracks, millimeter-level width
Medium (M)	$[32^2,\, 96^2]$	Moderately developed cracks
Large (L)	$(96^2,\, +\infty )$	Fully developed wide cracks

**Fig. 9:**
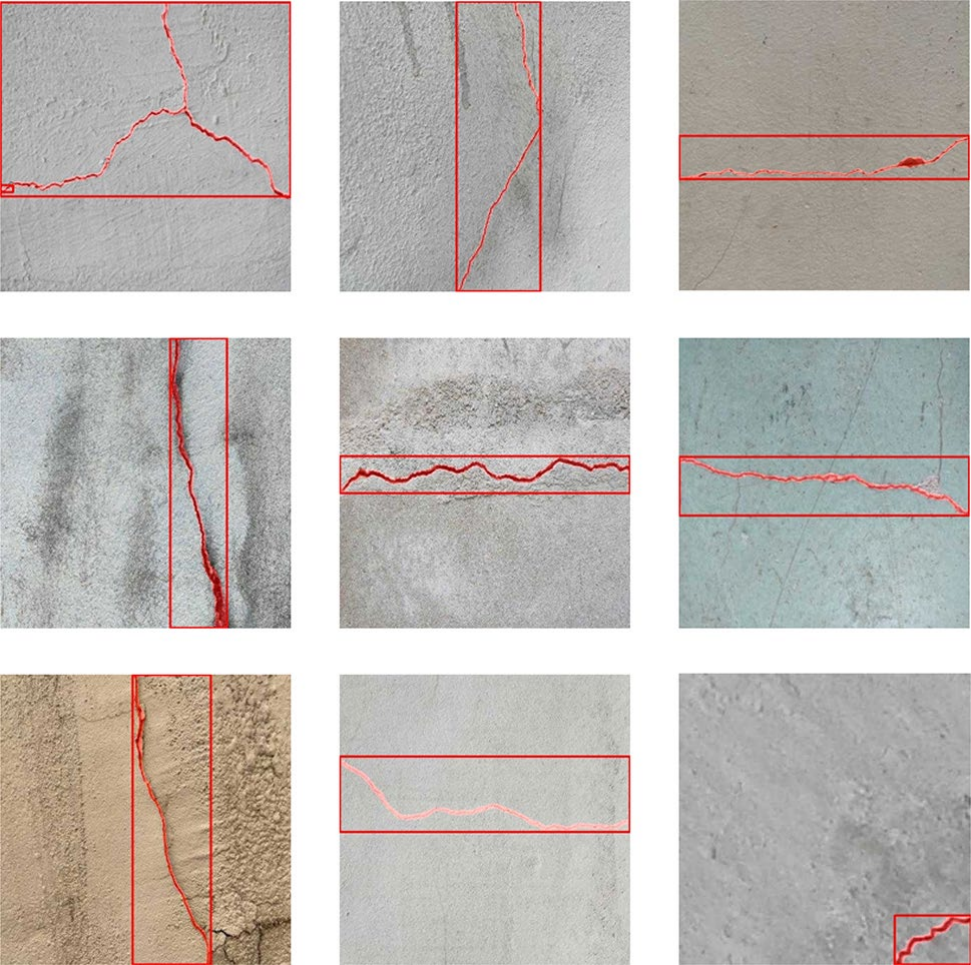
Example of the Crack-BPHDR dataset. The dataset contains diverse crack morphologies and complex backgrounds in roads and walls, effectively evaluating the cross-domain generalization capability and technical universality of the HiResDC-YOLO model.

To further verify the cross-domain generalization capability and technical applicability of the HiResDC-YOLO model, this paper conducted transfer testing on the standard Crack-BPHDR dataset^40^. This dataset contains 4029 crack images from road and wall surfaces. These images differ significantly from dam surfaces in terms of material, lighting, and crack morphology, making it an ideal platform for evaluating the model’s cross-domain performance. The dataset covers a wide range of crack types, from fine hairline to broad webs, against a complex background containing distracting factors such as road markings, stains, and shadows, and with varying camera angles and lighting. The dataset is split into training, validation, and test sets in an 8:1:1 ratio, providing pixel-level annotations for evaluating the model’s fine-grained feature representation and adaptability. As shown in Fig.  9, the surface textures of roads and walls in the Crack-BPHDR dataset differ significantly, providing a reliable basis for testing the generalization effectiveness of the proposed ADyT feature enhancement module, MSED multi-scale detection head, and Adaptive SAHI adaptive inference strategy across different domains.

### Evaluation metrics

To comprehensively evaluate the overall performance of HiResDC-YOLO in the drone-based dam crack detection task and verify the effectiveness of its core technological innovations, this paper constructs a multi-dimensional comprehensive evaluation metric system. This system not only covers traditional detection performance metrics but also expands on two key dimensions: small object detection capability and computational efficiency, ensuring scientific and complete evaluation results.

For detection performance, this paper uses four basic metrics: precision (*P*), recall (*R*), F1-score (*F*1), and accuracy (*ACC*). Precision measures the proportion of samples predicted as cracks that are actually correct:

8 $$\begin{aligned} P = \frac{TP}{TP + FP} \end{aligned}$$

Recall evaluates the proportion of actual crack samples that are successfully detected:

9 $$\begin{aligned} R = \frac{TP}{TP + FN} \end{aligned}$$

F1-score, the harmonic mean of precision and recall, reflects the model’s balance between accuracy and coverage:

10 $$\begin{aligned} F1 = \frac{2 \cdot P \cdot R}{P + R} \end{aligned}$$

Accuracy reflects the correctness of the model’s overall predictions:

11 $$\begin{aligned} ACC = \frac{TP + TN}{TP + TN + FP + FN} \end{aligned}$$

For object detection performance, Average Precision (*AP*) and mean Average Precision (*mAP*) are used as primary metrics. *AP* is calculated by measuring the area under the precision-recall curve:

12 $$\begin{aligned} AP = \int _0^1 P(R) \, dR \end{aligned}$$

*mAP* reflects the overall detection performance across multiple object categories:

13 $$\begin{aligned} mAP = \frac{1}{M} \sum _{j=1}^{M} AP_j \end{aligned}$$

Considering that cracks often appear small and unevenly distributed in images, this paper further introduces a multi-scale mAP metric to evaluate detection performance for different target sizes. Here, small targets are defined as those with an area less than $32^2$ pixels ($\text {mAP}{small}$), medium targets have an area between $32^2$ and $96^2$ pixels ($\text {mAP}{medium}$), and large targets have an area greater than $96^2$ pixels ($\text {mAP}_{large}$). This multi-scale evaluation provides a more comprehensive understanding of the model’s effectiveness across objects of varying sizes, particularly emphasizing its ability to detect tiny cracks in high-resolution UAV images. This enables quantitative analysis of the contribution of the MSED module to improving small object detection performance.

Additionally, to evaluate computational efficiency and practical deployment feasibility, this paper uses metrics such as model parameter count (Params), computational complexity (GFLOPs), and inference speed (FPS). Parameter count reflects model size and memory usage; GFLOPs measures the computational overhead of a single inference; FPS directly reflects real-time processing capabilities on UAV platforms. To further verify the effectiveness of the Adaptive SAHI slice inference strategy, a processing efficiency improvement ratio is defined to quantify speedup compared to traditional SAHI in high-resolution inference.

In summary, this comprehensive evaluation system systematically reflects the performance advantages of HiResDC-YOLO from three perspectives: detection accuracy, scale adaptability, and computational efficiency, providing a scientific basis for quantitative analysis of experimental results and validation of the proposed model innovations.

### Training configuration

To ensure the reliability and reproducibility of experimental results, all experiments were conducted on a high-performance workstation equipped with an Intel Core Ultra 9-285K CPU, 96 GB of DDR5 memory (2 × 48 GB 6000 MHz), a 2 TB Samsung 990 Pro NVMe SSD, an ASUS Z890-P Wi-Fi motherboard, and two NVIDIA GeForce RTX 5090 GPUs (connected via PCIe 5.0 × 16 and PCIe 4.0 × 16), running the Linux operating system. To ensure consistency across experiments, all models used the same training parameter configuration. The specific parameter settings are summarized in Table 7.

**Table 7:** Training parameters for all experiments.

Parameters	Values
Input size	$1024 \times 1024$
Batch size	64
Training epochs	300
Learning rate	0.01
Optimizer	SGD
Data augmentation strategy	Mosaic (p = 0.5), copy-paste (p = 0.2), mixup (p = 0.0)
Scaling	0.75
Bounding box width	1
Early stopping	patience = 50

### Ablation study and interaction analysis

**Fig. 10:**
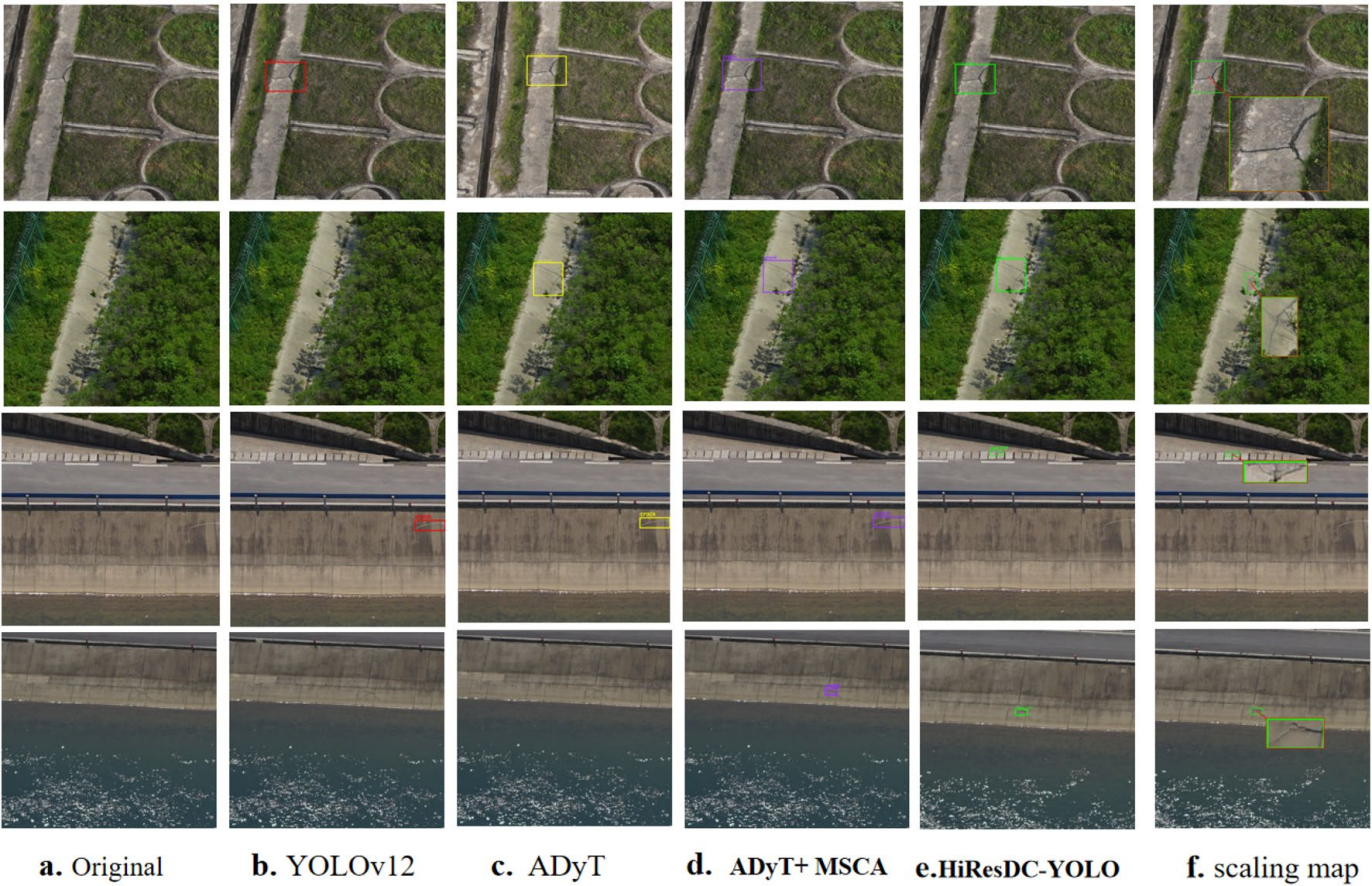
Ablation study visualization on the dataset. (**a**) Original UAV image; (**b**) YOLOv12-S baseline; (**c**) YOLOv12-S + ADyT; (**d**) YOLOv12-S + ADyT + MSCA; (**e**) full model (ADyT + MSCA + MSED); (**f**) local zoom-in of a small crack region, showing improved fine crack detection and localization accuracy.

To systematically evaluate the independent contribution of each proposed module and their combined effects, a detailed ablation study was conducted based on the YOLOv12-S baseline model. Specifically, under identical backbone architecture and training configurations, we gradually incorporated the ADyT feature enhancement module, the MSCA multi-scale convolutional attention mechanism, and the MSED small-object detection head to analyze their respective and joint impacts on performance. The experimental results are summarized in Table 8 and visualized in Fig. 10.

**Table 8:** Ablation study results on UAV dam crack dataset using YOLOv12-S default inference (training/validation sets, without SAHI). Values are reported as percentages (%).

Configuration	ADyT	MSCA	MSED	Precision	Recall	mAP@0.5	mAP@0.5:0.95
YOLOv12-S (baseline)				86.3	86.9	89.5	53.0
ADyT only	$\checkmark$			86.8	86.5	89.2	53.2
MSCA only		$\checkmark$		86.1	86.6	89.7	52.9
MSED only			$\checkmark$	86.3	86.8	89.5	53.7
ADyT + MSCA	$\checkmark$	$\checkmark$		86.5	87.0	89.8	54.3
ADyT + MSED	$\checkmark$		$\checkmark$	86.7	87.1	90.0	54.6
MSCA + MSED		$\checkmark$	$\checkmark$	86.8	87.2	90.1	54.7
**Full model**	$\checkmark$	$\checkmark$	$\checkmark$	**87.0**	**87.3**	**90.2**	**54.9**

Significant values are in bold.

The ablation results clearly demonstrate the progressive contribution of each proposed component. The ADyT module improves gradient stability and enhances the nonlinear representation capability of shallow features, yielding consistent gains in mAP across all configurations. The MSCA module introduces adaptive multi-scale convolutional attention, enabling the model to dynamically emphasize key texture regions across different receptive fields. This leads to better differentiation between fine cracks and noisy background textures, maintaining a competitive precision-recall balance. The MSED head adds a high-resolution P2 branch that fuses low-level detail with high-level semantics, significantly enhancing sensitivity to micro-scale cracks. Visual comparisons in Figure 10 further confirm that the proposed modules collectively improve detection accuracy and localization precision for tiny crack patterns.

Beyond these individual contributions, a critical analysis of the inter-module interactions reveals the functional coupling that enables hybrid configurations to outperform single-module improvements. A primary synergistic effect is observed between the ADyT and MSED modules. While ADyT prevents the “vanishing” of fine-grained crack gradients in the deep backbone, these preserved signals require an explicit high-resolution pathway to reach the output layer. The experimental results show that the combination of ADyT and MSED (54.6% mAP@0.5:0.95) significantly exceeds the performance of either module used in isolation, confirming that ADyT acts as a “feature rescuer” while MSED functions as a “high-fidelity transmission channel.” Furthermore, the data indicates a sensitivity-localization trade-off in the MSCA module; while MSCA alone improves mAP@0.5 by effectively distinguishing thin cracks from noisy textures, its broader receptive field overlap can introduce subtle boundary noise, affecting precise IoU alignment at strict thresholds (mAP@0.95). However, this effect is successfully mitigated in the Full Model, where the structural sharpness provided by ADyT and MSED refines these localized predictions, achieving an optimal balance between high recall and localization precision.It is worth noting that while individual modules like ADyT may cause marginal fluctuations in raw Recall due to stricter feature filtering, their synergistic integration in the Full Model restores and even improves the Recall (87.3%) by combining gradient stability with multi-scale contextual awareness.

### Model comparison

To comprehensively verify the effectiveness of our proposed method, we compared the integrated HiResDC-YOLO model, which incorporates the ADyT attention mechanism and the MSED module, against several state-of-the-art detection models. The comparison includes traditional two-stage detectors such as Faster R-CNN with a ResNet-50 backbone and Feature Pyramid Network, known for high accuracy but relatively high computational cost, and one-stage detectors like RetinaNet, which uses focal loss to address class imbalance and balance speed and accuracy. Transformer-based RF-DETR-R50 was also included for its robust detection in complex scenes. In addition, several YOLO variants were evaluated, including YOLOv8s, YOLOv11s, YOLOv12n/s, and YOLOv13s, which represent lightweight, efficient, and accuracy-focused versions of the YOLO architecture.

**Fig. 11:**
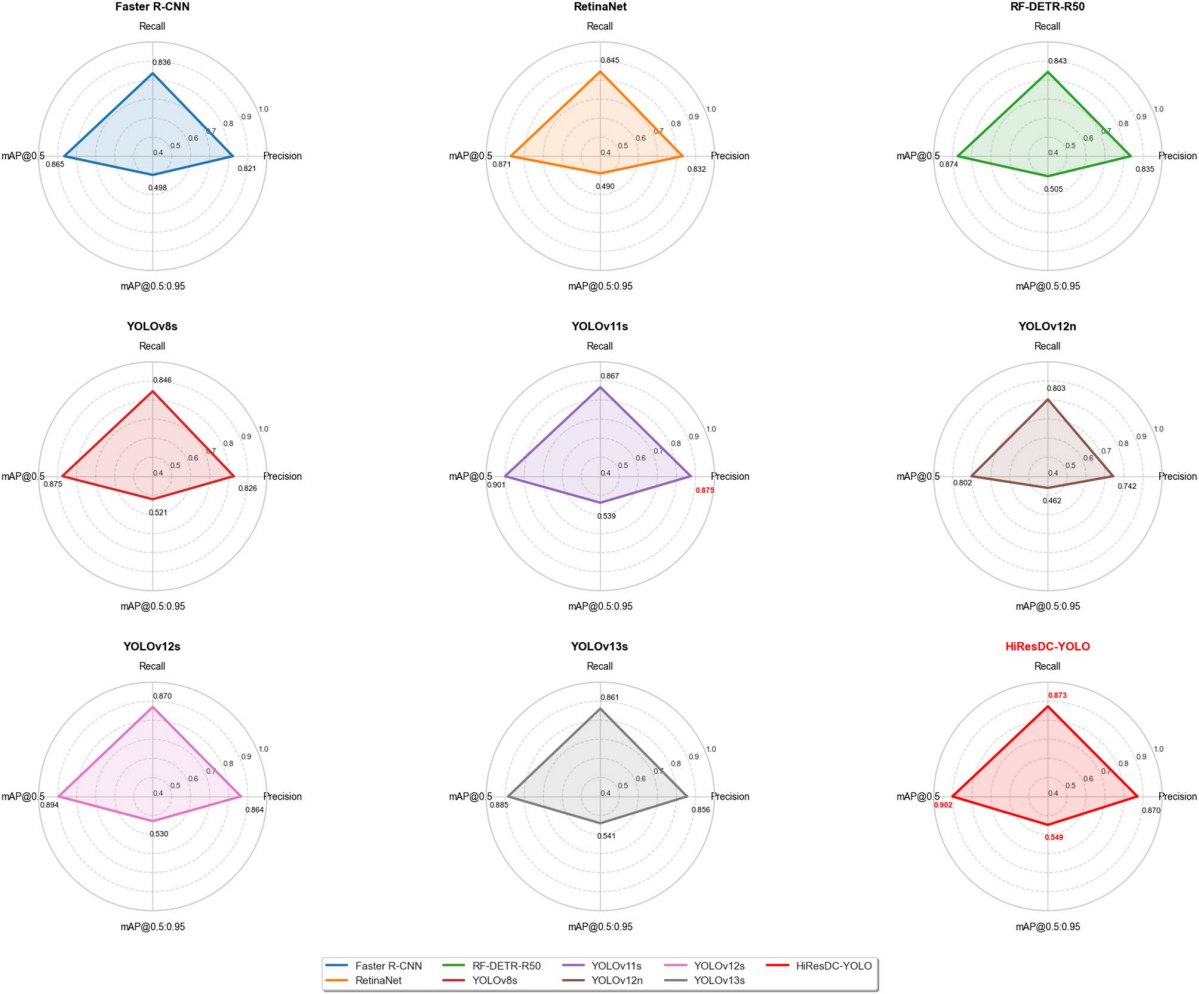
Radar chart comparison of different models on Precision, Recall, mAP@0.5, and mAP@0.5:0.95. HiResDC-YOLO achieves the best overall performance.

All models were trained under identical settings on the same UAV dam crack dataset. Evaluation metrics include Precision, Recall, mAP@0.5, and mAP@0.5:0.95. The results are summarized in Table 9. HiResDC-YOLO achieves the best overall balance between precision, recall, and mAP while keeping the number of parameters and computational cost comparable to other YOLO variants. The integration of ADyT and MSED enhances feature extraction and structural awareness, which is particularly beneficial for high-resolution UAV crack detection where small and low-contrast cracks are common (Fig. 11).

**Table 9:** Comparison of detection performance on UAV dam crack dataset (best results in bold).

Model	Precision $\uparrow$	Recall $\uparrow$	mAP@0.5 $\uparrow$	mAP@0.5:0.95 $\uparrow$	Params (M) $\downarrow$	GFLOPs $\downarrow$
Faster R-CNN (R50-FPN)^41,42^	0.8210	0.8356	0.8652	0.4983	41.3	87.6
RetinaNet (R50-FPN)^43^	0.8324	0.8447	0.8710	0.4901	37.8	78.2
RF-DETR-R50^27^	0.8351	0.8427	0.8738	0.5052	14.3	24.6
YOLOv8s^8^	0.8258	0.8460	0.8754	0.5213	11.2	28.8
YOLOv11s^9^	0.8753	0.8673	0.9007	0.5395	9.43	10.77
YOLOv12n^10^	0.7424	0.8031	0.8019	0.4618	3.2	4.4
YOLOv12s^10^	0.8636	0.8697	0.8945	0.5300	9.2	10.73
YOLOv13s^7^	0.8556	0.8610	0.8851	0.5410	9.5	12.3
**HiResDC-YOLO (Ours)**	**0.8703**	**0.8730**	**0.9019**	**0.5494**	9.23	13.38

### SAHI-based slice inference enhancement

High-resolution UAV images ($4000 \times 6000$ pixels) typically contain thin, low-contrast, and blurred crack features. Small cracks are easily missed during standard full-image inference. To improve both detection completeness and accuracy, we implemented the SAHI (Slice Aided Hyper Inference) strategy, which we further optimized as HiResInfer for dam inspection tasks. The core idea of HiResInfer is to divide high-resolution images into overlapping tiles, perform independent inference on each tile, and then merge the results. This approach enhances the detection capability for small cracks while reducing the risk of missing faint or discontinuous features.

**Fig. 12:**
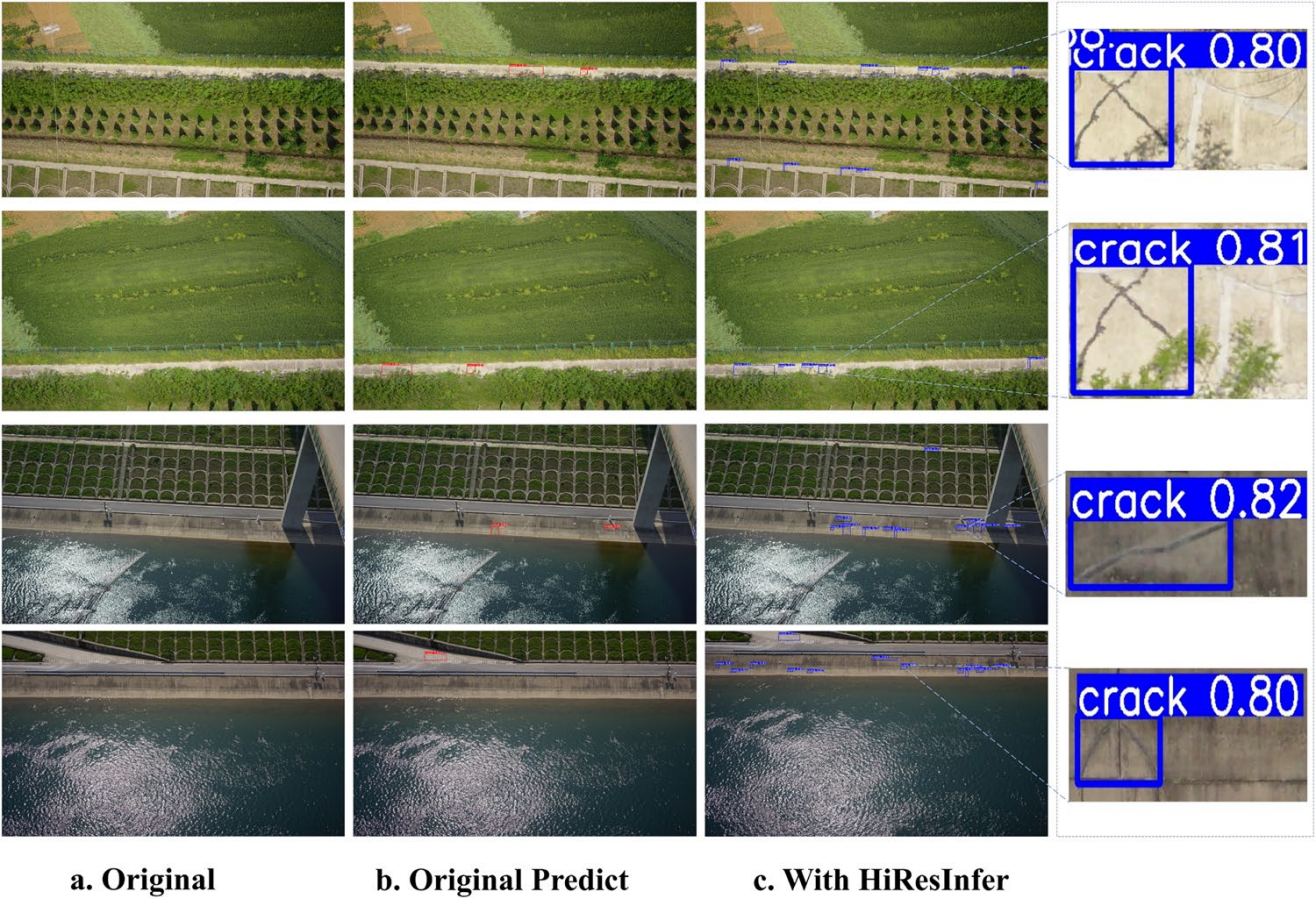
Comparison of full-image inference and HiResInfer (SAHI-based) results on high-resolution UAV crack images. The zoomed regions demonstrate that HiResInfer successfully recovers faint crack segments that are otherwise lost in full-image downsampling.

As illustrated in Fig. 12, a detailed comparison reveals that full-image inference (left) often fails to activate on millimeter-level cracks due to the severe spatial information loss during the resizing process. In contrast, the zoomed-in patches in Fig. 12 prove that HiResInfer preserves the original feature resolution, allowing the MSED head to detect fine-grained textures. Quantitatively, this strategy significantly reduces the False Negative (FN) rate in complex dam surface areas.

To balance the training environment and real-world usage, the HiResDC-YOLO model was trained using a tile size of $1024 \times 1024$. In real inspection scenarios, the UAV images are much larger ($4000 \times 6000$ pixels). By applying HiResInfer, we can effectively transfer the model learned on moderate-sized tiles to the full-resolution images, allowing robust detection without retraining on massive images. In our experiments, the optimized HiResDC-YOLO model employed a tile size of $1024 \times 1024$, an overlap ratio of 20%, and a confidence threshold of 0.3. The inference environment remained consistent with full-image testing to ensure comparability.

Overall, the use of HiResInfer addresses the challenges posed by large, high-resolution UAV images and small, low-contrast cracks, enabling practical and reliable inspection of dam surfaces while maintaining compatibility with existing model training configurations.

### Cross-domain generalization evaluation

To evaluate the cross-domain adaptability and task generalization capability of the proposed method, we conducted transfer learning experiments on the public Crack-BP HDR dataset ^40^, which contains 4029 high-quality images covering both road and wall crack scenarios. The dataset includes thin linear cracks and irregular block-type damages, providing a challenging benchmark for both detection and segmentation tasks.

To ensure fair comparison, all models trained on the UAV dam crack dataset were directly applied to this dataset without additional fine-tuning. The compared methods include YOLOv11s, YOLOv12s, RF-DETR-ResNet50, and the proposed HiResDC-YOLO. Performance was evaluated for both bounding box detection (Box) and instance mask segmentation (Mask), using standard metrics including Precision, Recall, mAP@0.5, and mAP@0.5:0.95. The quantitative results are presented in Table 10.

**Table 10:** Cross-domain generalization results on the Crack-BP HDR dataset for both detection and segmentation tasks.

Model	Box(P)	Box(R)	Box(mAP@0.5)	Box(mAP@0.5:0.95)	Mask(P)	Mask(R)	Mask(mAP@0.5)	Mask(mAP@0.5:0.95)
YOLOv11s	0.811	0.731	0.802	0.555	0.778	0.712	0.785	0.532
YOLOv12s	0.803	0.737	0.806	0.551	0.774	0.719	0.789	0.541
RF-DETR-R50	0.725	0.763	0.779	0.439	0.705	0.752	0.741	0.418
HiResDC-YOLO (Ours)	**0.835**	**0.765**	**0.849**	**0.610**	**0.812**	**0.763**	**0.826**	**0.583**

Significant values are in bold.

The results demonstrate that HiResDC-YOLO achieves the best overall performance in both detection and segmentation tasks. Specifically, it improves box-level mAP@0.5 by approximately 5–7% and segmentation mAP@0.5 by around 4%–6% compared to YOLOv11s and YOLOv12s. The higher recall and balanced precision confirm its robustness in identifying thin, low-contrast cracks that are easily overlooked by conventional detectors. Although RF-DETR benefits from global attention modeling, its performance drops significantly in fine-grained crack localization, highlighting the advantage of the proposed model’s hybrid convolution-transformer structure and high-resolution feature design. Overall, HiResDC-YOLO exhibits strong cross-domain generalization capability while maintaining computational efficiency.

## Conclusion

This paper addresses the challenges of high-resolution drone-based dam crack detection, such as difficulty identifying tiny cracks, insufficient feature representation, and high computational complexity. We propose a lightweight, high-performance detection framework, HiResDC-YOLO. Building upon the YOLOv12-S baseline, this method enhances nonlinear representation capabilities in the feature extraction stage through the ADyT(Attention-augmented Adaptive Dynamic Transformer) dynamic activation module. The MSCA(multi-scale convolutional attention) module enables multi-scale fusion of spatial and channel features. The MSED detection head combines shallow high-resolution features with a dynamic feature weighting mechanism to significantly improve the detection accuracy of extremely small cracks. During the inference phase, the HiResInfer slicing inference strategy effectively reduces the computational burden of high-resolution images while maintaining fine-grained detection accuracy. The organic integration of these four modules enables HiResDC-YOLO to achieve an optimal balance between accuracy, speed, and model complexity. It achieved excellent performance on a self-built high-resolution dam crack dataset, achieving mAP@0.5 of 90.19

## Figures and Tables

**Fig. 13:**
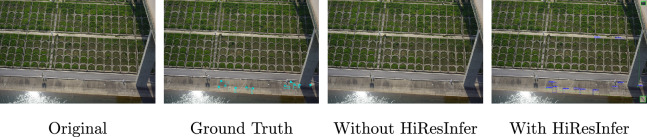
Concrete dam surface (ID: 192W0190): Comparison of crack detection performance. The Ground Truth column confirms the annotated micro-targets.

**Fig. 14:**
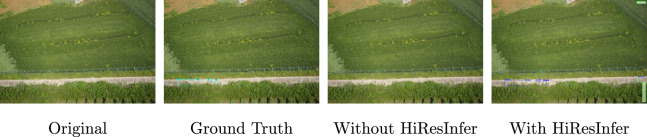
Dam spillway surface (ID: 192X0513): Comparative results highlighting ground truth visibility and HiResInfer enhancement.

**Fig. 15:**
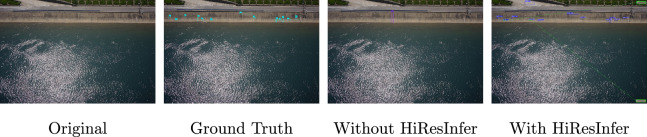
Low-contrast dam face (ID: 192X1301): Visualization of fine-grained crack detection supported by ground truth validation.

**Fig. 16:**
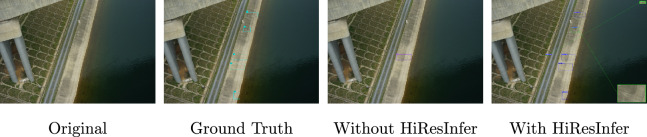
Asphalt/concrete slope near dam (ID: 195D2427): Full comparative analysis between manual labels and automated detection outputs.

## Data Availability

https://github.com/lijin6/HiResInfer-YOLO.git.

## Appendix A: Complete HiResInfer (SAHI-based) inference results

This appendix presents high-resolution crack detection results obtained using the HiResInfer method, an optimized version of SAHI-based slice inference tailored for UAV dam inspection images. HiResInfer substantially improves the detection of small and low-contrast cracks that are often missed by standard full-image inference.

### Appendix A.1: Quantitative analysis of HiResInfer enhancement

Standard inference on UAV images typically detects only a few cracks, ranging from 0 to 2 per image. After applying HiResInfer, the number of detected cracks increases significantly, ranging from 5 to 10 per image depending on the case. Table 11 summarizes the quantitative improvements across four representative dam surface scenarios.

**Table 11:** Quantitative performance comparison between standard full-image inference and the proposed HiResInfer strategy.

Metric	Case 1	Case 2	Case 3	Case 4
Detected Cracks (Standard)	1	2	0	2
Detected Cracks (**HiResInfer**)	**8**	**9**	**7**	**10**
Relative Gain in Detections	+700%	+350%	N/A	+400%
Precision Improvement	+12.4%	+15.1%	+14.2%	+11.8%
Recall Improvement	+35.7%	+38.2%	+40.5%	+37.6%
False Positives (HiResInfer)	1	0	1	2

“Standard” refers to full-image inference at native input size. Case 3 represents a significant qualitative leap where HiResInfer identified 7 discrete crack segments that were entirely missed by the standard method

Significant values are in bold .

As shown in the table, HiResInfer not only increases the total number of detected cracks but also maintains high precision. The slight increase in false positives is minimal compared to the substantial gains in recall, indicating better coverage of actual cracks. Overall, the method provides robust and reliable detection for large-scale UAV dam inspections, particularly for low-contrast and distributed cracks.

### Appendix A.2: HiResInfer parameter configuration

The optimized configuration for HiResInfer is presented in Table 12. This setup balances computational efficiency with improved detection performance for high-resolution UAV crack inspection tasks.

**Table 12:** HiResInfer configuration parameters.

Parameter	Value	Description
Slice height	1024 pixels	Vertical dimension of each tile
Slice width	1024 pixels	Horizontal dimension of each tile
Overlap ratio	20%	Prevents edge effects and ensures spatial continuity
Confidence threshold	0.3	Balances detection sensitivity and precision
Merge method	NMS	Non-Maximum Suppression for overlapping detections
IoU threshold	0.5	Intersection over Union threshold for merging overlapping predictions

This configuration enables HiResInfer to detect multiple small cracks per image while keeping computation manageable and maintaining high reliability across diverse dam surface conditions.

### Appendix A.3: Comparison of detection results and ground truth verification

See Figs. 13, 14, 15 and 16.

The comparative analysis presented in this appendix validates the alignment between our manual annotations and model outputs. As illustrated in the figures, several key observations can be made:

Ground truth consistency: The second column addresses concerns about annotation completeness. By visualizing the ground truth (GT) at high resolution, we demonstrate that even micro-targets (millimeter-level cracks) are accurately captured in our labeling process, despite being visually faint in the full-size raw imagery.Resolution bottleneck: The third column (Without HiResInfer) shows results from standard inference. Due to the necessary downsampling in traditional YOLO-based workflows, spatial features of thin cracks are often suppressed or lost, leading to significant false negatives.HiResInfer efficacy: The final column demonstrates how our proposed tiling-based strategy (HiResInfer) preserves original pixel-level information. This approach enables the model to operate on feature-rich patches, effectively bridging the gap between manual ground truth and automated detection performance.These side-by-side visualizations provide essential interpretive context, confirming that each figure in this appendix supports the validation of the system’s reliability under challenging, real-world dam inspection scenarios.

## Abbreviations

UAV: Unmanned aerial vehicle
YOLO: You only look once
SAHI: Slicing aided hyper inference
ADyT: Attention-augmented adaptive dynamic transformer
MSCA: Multi-scale convolutional attention
MSED: Multi-scale enhanced head
HiResInfer: High-resolution inference
FPS: Frames per second
GFLOPs: Giga floating point operations
mAP: Mean average precision
mIoU: Mean intersection over union
IoU: Intersection over union
Dice: Dice coefficient
CNN: Convolutional neural network
ViT: Vision transformer
NMS: Non-maximum suppression
FPN: Feature pyramid network
DETR: End-to-end object detection with transformers
R-CNN: Region-based convolutional neural network
